# Exercise in Isolation- A Countermeasure for Electrocortical, Mental and Cognitive Impairments

**DOI:** 10.1371/journal.pone.0126356

**Published:** 2015-05-11

**Authors:** Vera Abeln, Eoin MacDonald-Nethercott, Maria Francesca Piacentini, Romain Meeusen, Jens Kleinert, Heiko K. Strueder, Stefan Schneider

**Affiliations:** 1 Institute of Movement and Neurosciences, German Sport University Cologne, Cologne, Germany; 2 Cambridge University’s Hospitals NHS Trust, Cambridge, United Kingdom; 3 Institute Paul Emile Victor, Brest, France; 4 University of Rome Foro Italico, Rome, Italy; 5 Faculty of Physical Education and Physiotherapy, Department of Human Physiology, Brussels, Belgium; 6 School of Public Health, Tropical Medicine and Rehabilitation Sciences, James Cook University, Townsville, Queensland, Australia; 7 Institute of Psychology, Department of Health and Social Psychology, German Sport University Cologne, Germany; 8 Faculty of Science, Health, Education and Engineering, University of the Sunshine Coast, Maroochydore, Queensland, Australia; Purdue University, UNITED STATES

## Abstract

**Introduction:**

Mental impairments, including deterioration of mood and cognitive performance, are known to occur during isolation and space missions, but have been insufficiently investigated. Appropriate countermeasures are required, such as exercise, which is known to prevent mood disorders for prolonged space and isolation missions. Based on the interaction of brain activity, mood and cognitive performance, this study aims to investigate the effect of long-term isolation and confinement and the long-term effect of exercise on these parameters.

**Methods:**

Eight male volunteers were isolated and confined for about eight month during the winter period at the Antarctic Concordia Station. Every six weeks electroencephalographic measurements were recorded under rest conditions, and cognitive tests and a mood questionnaire were executed. Based individual training logs, subjects were afterwards separated into an active (> 2500 arbitrary training units/interval) or inactive (< 2500 arbitrary training units/interval) group.

**Results:**

A long-term effect of exercise was observed for brain activity and mood. Regularly active people showed a decreased brain activity (alpha and beta) in the course of isolation, and steady mood. Inactive people instead first increased and than remained at high brain activity accompanied with a deterioration of mood. No effect of exercise and isolation was found for cognitive performance.

**Conclusion:**

The findings point out the positive effect of regularly performed voluntary exercise, supporting subjective mental well-being of long-term isolated people. The choice to be regularly active seems to support mental health, which is not only of interest for future isolation and space missions.

## Introduction

At a time when prolonged space missions are being considered, there is a growing need for preparative investigations. Psychophysiological impairments in terms of deterioration in mental health (mood and cognition, WHO) are known to be associated with long duration space missions, but are not well understood [1–4]. Nevertheless stable psychological well-being and cognitive function are vital for the success and safety of such missions, and appropriate countermeasures will be necessary.

Over-wintering in the interior of Antarctica provides an environment that parallels the conditions of isolation, confinement and stress likely to be faced by a crew on a long-duration space mission, and is therefore considered to provide an suitable analog for investigating psycho-physiological changes [5].

Studies of polar expeditions report that winter crew experience symptoms similar to those experienced by crew in space, such as sleep disruption, impaired cognitive performance, negative affect, interpersonal tension, and conflict resulting in increased sensitivity to physical and social cues (for review see [6]. Proposed reasons for these various symptoms experiencing polar or space expeditions are manifold, such as the lack of sunlight, the hostile environment, the loneliness, altered daily behavior including food and physical activity, sensory deprivation, the confined and restricted social interaction with crewmates, with concurrent isolation from ‘normal’ society. Sensory deprivation is thought to be associated with reductions in the brain’s cortical activity [7]. Symptoms often become more severe around the third quarter of isolation [8] or at the mid-point of isolation, with some alleviation towards the end [9]. Medications can be used to treat the sleep and mood problems crews have experienced, but it would generally be preferable to find treatments to that avoided the need for drugs [9].

It is known and generally accepted that exercise has positive influences on several of the symptoms associated with isolation, such as mood, cognition and mental well-being [10–13]. In fact, there are physiological and psychological models to explain the relationship between exercise and mood; implicating hormonal, neuro-chemical, neuro-electrical, thermoregulatory or state-related (self-efficacy, distraction) changes [10, 14–17]. While the underlying mechanisms are not fully understood, most of these theories involve the participation of the brain. Moreover, exercise has been shown to counteract psycho-physiological deconditioning during long-term isolation mediated by altered brain activity [7, 18].

Exercise, therefore, seems to be a potential countermeasure for psychological and mental deconditioning, as well as for the recognized effects that microgravity has on the musculoskeletal and cardiovascular systems during isolation and space missions [1–4]. However, evidence is still sparse, due to the fact that space and isolation missions are rare and involve small numbers of participants. Before implementing specific exercise interventions for such purposes during such missions, further proof is needed.

The European Space Agency (ESA) uses Concordia Station in Antarctica as an analogue for space, coordinating a human research protocol on site each year. Concordia Station is a permanent, joint French-Italian research station crewed all year round. It is operated by the Institute Polaire Français Paul Emile Victor and the Programma Nazionale Di Ricerche in Antartide. It is built 3233m above sea level on the Antarctic plateau Dome C. It is located 950 km away from the nearest coast, 1670 km away from the South Pole and 560 km away from the nearest neighboring station. During the nine-month period defined as winter (February to November), the location’s temperature is a mean -65°C [19], too cold for vehicles to reach the station, and as a result the station is completely physically isolated. Due to isolation and limited possibility to go outside the station, life at Concordia in winter-term is more monotone compared to summer-term, when more people and work to prepare for winter is present. At midwinter, there is no sunrise for approximately three months and in that time, there is a seven-week period of continuous darkness. Since 2005 Concordia has been crewed through the winter, each year by a team of around 10–14, comprised of physical scientists and technical personnel.

This study aimed to investigate the effect of long-term isolation at the Antarctic Concordia Station on human psycho-physiological state including mood, cognitive performance and brain cortical function. It is hypothesized that in an isolated group, physically active individuals exercising voluntarily and frequently with certain intensity would benefit, and therefore show less deterioration in mood and cognitive performance, accompanied by altered brain cortical activity.

## Materials & Methods

### Participants and Procedures

The study was conducted during the winter-over in 2011 (DC7) at the Antarctic research station Concordia. Before traveling to Antarctica, all fourteen members of the all-male winter-over crew were informed about the content of the study and participated in an information session. Thirteen members of the crew agreed to voluntarily participate in our study and signed a written consent form approved by the ethics committees of both the European Space Agency (ESA) and the Institute Polaire Paul Emile Victor (IPEV). A familiarization session for was implemented at the station in the three weeks prior to the commencement of the winter period.

During the period of isolation, volunteers performed the tests once every six weeks (interval). One crewmember was tested per day, during a two-week period. The tests consisted of a rest-EEG recording, a mood questionnaire and three cognitive tasks (see below). For ten individuals the measurements were made during a session that started at 3 pm. Three of the participants worked nightshift hours and they were tested after their midday meal, approximately seven hours after awakening. The measurements were all recorded in the same laboratory under the same conditions.

Participants were free to exercise in the gym on a voluntarily basis at any time. They were asked to fill out a training log containing the mode, duration and the rating of perceived exertion (RPE) measured on visual analogue scales.

**Fig 1:**
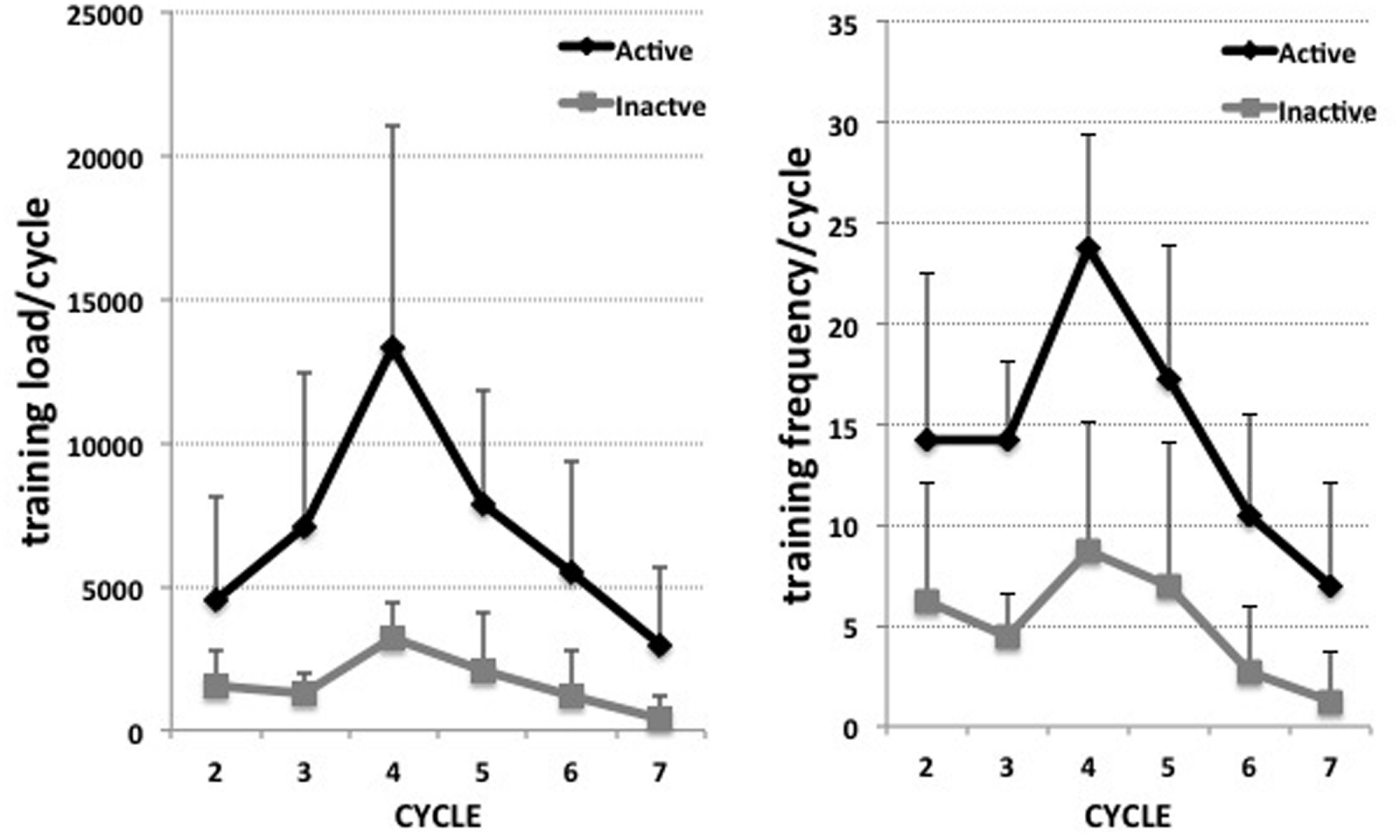
Training load and frequency. Training load (left) and training frequency (right) per interval for the active (black) and inactive (grey) group for intervals 2 to 7 (interval 1 data missing). Displayed are mean values plus/minus standard deviations.

Eight of the crew completed our protocol and were categorized afterwards to either the active group (G_act_, n = 4, mean age: 42.3± 8.8 years, height: 180.25± 8.1 cm, weight: 80.25) or the inactive group (G_inact_, n = 4, mean age: 37± 5.6 years, height: 170± 6,4 cm, weight: 64,8± 5.3) based on the training load data. The criteria for the differentiation between the two groups were based on the amount of training/interval. The G_act_ was considered to be those individuals who had training load averaging higher than 2500 arbitrary training units load/interval (training load = RPE *volume (min)). The session RPE (or training load per session) is a validated method to monitor training load in different sports, including strength training [20]. How groups differ in training load and training frequency is displayed in Fig 1. It should be mentioned that one out of the four members of G_inact_ trained with a frequency comparable to G_act_, but with far lower training load.

### EEG

Electroencephalography (EEG) was used for recording brain cortical activity for five minutes sitting in a relaxed position with eyes closed, about 30 min after arriving at the laboratory. The electrode cap (ActiCap EEG Active Electrode System, Brain Products GmbH, Gilching, Germany) with 32 active Ag/AgCl electrodes was fitted to their head. Electrodes Fp1, Fp2, F7, F3, Fz, F4, F8, FC5, FC1, FC2, FC6, T7, C3, Cz, C4, T8, TP9, CP5, CP1, CP2, CP6, TP10, P7, P3, Pz, P4, P8, PO9, O1, Oz, O2 and PO10 were placed according to the 10–20 system [21]. A reference electrode was integrated in a triangle of FP1, FP2 and FZ. EEG data was collected and recorded using a Brain Vision Amplifier and RecView software (Brain Products GmbH, Munich, Germany) at a sample frequency of 500 Hz. The cap was breathable to avoid heat accumulation and fixed using an elastic chinstrap to prevent shifting of the electrodes. Each electrode was filled with electrode gel (Super-Visc, EasyCap GmbH, Herrsching, Germany) via a syringe with a blunt-tipped needle, which was used to inject the gel into to the electrodes on the subject’s head and to move away the hair underneath the electrodes to facilitate signal transduction. Alpha activity (frequency range between 7.5 and 12.5 Hz) traditionally is assumed to reflect relaxation, whereas beta activity (12.5 to 35 Hz) was seen as an indicator of arousal; known as the model of cortical arousal [22]. More recently, numerous studies have demonstrated parallel changes within the alpha and beta frequency band [7, 23, 24], contradicting this theory, and call for a more careful interpretation. Here, we used the traditional division for comparison with former studies, but for the discussion we will take the current findings into account.

Electrodes with impedance values exceeding 10 kΩ were excluded from further analysis. Data was filtered (Brain Vision Analyzer software, Brain Products, Munich, Germany) with Butterworth zero phase filters including a notch-filter at 50 Hz. Low cut-off was set at 3 Hz and high cut-off at 70 Hz, with a time constant of 0.053 seconds and 48 dB/oct. Noisy channels were replaced using the topographic interpolation function (interpolation type: spline, order: 4, degree: 10, Lambda: 1E-05). The five-minute recordings were divided into four-second segments with 10% overlap. An automatic artifact correction algorithm was applied, which allowed a maximum voltage step of 50 μV per data sample, maximal difference of values in intervals 200 μV with an interval length of 200 ms, an amplitude range of -200 to 200 μV, and 0.5 μV lowest activity in intervals with an interval length of 200 ms. Identified artifacts were marked and the data from 200 ms before to 200 ms after the event were automatically removed. In addition, all data were visually checked and corrected for movement, pulse-related and myogenic artifacts. Data were then baseline corrected based on a four-second interval and Fast Fourier Transformation (power spectrum, hanning window 10%, resolution 0.244 Hz). All remaining segments were averaged and pooled over all electrodes to reveal the mean global activity, which was exported as raw sum values for the alpha (7.5–12.5 Hz) and beta (12.5–35 Hz) frequency band.

Natural logarithms (LN) were applied to the raw data in order to diminish individual differences in cortical activity and to improve comparability. Data from the 2^nd^ to the final (7^th^) intervals were calculated as percentage changes from the first interval for the alpha and beta frequency range respectively.

### Mood

Following the EEG recording, participants completed a paper-and-pencil mood questionnaire. The questionnaire consisted of two scales. The first scale (PEPS, Perceived Physical State) assessed the physical well-being of the participants. The PEPS comprises 20 adjectives divided into the sub-dimensions of perceived physical activation, perceived physical fitness, perceived physical flexibility and perceived physical health, which has been validated on a total of 645 people (Cronbach’s alpha intraclass correlation coefficient .82 and .92) for the assessment of short-term changes of mood in athletes [25]. The second scale measured the perceived psychological state and is a short form of the “Eigenzustandsskala” (EZ-K, which translates as “Personal State scale”) [26]. The EZ-K is comprised of 16 adjectives measuring perceived psychological strain and perceived motivational state. Psychological strain is divided into sub dimensions sleepiness, mood, calmness and recovery, and motivational state is divided into self-confidence, willingness to seek contact, social acceptance and readiness to strain).

The questionnaires were presented in the participants’ native language. Participants were instructed to rate each adjective in terms of how much it described their state at the time they were completing the questionnaire, on a scale from 0 (not at all) to 5 (absolutely). For analysis of negative adjectives the scale was reversed in order to be able to directly compare positive and negative adjectives, and analyze the scores. Adjectives were first pooled into their sub-dimension and afterwards averaged into the three dimensions (Psychological Strain—‘STRAIN’, Motivation—‘MOT’, Perceived Physical State—‘PEPS’). Data from intervals 2 to 7 were analyzed in terms of percentage changes from interval 1 for each dimension.

### Cognitive Tasks

Directly after the questionnaire, participants carried out three commercially available cognitive tasks (www.lumosity.com) on an Ipod touch (Apple Inc. Cupertino, California, USA): *Speed Match* which tests object recall, visuoperceptual speed and reaction time, *Chalkboard Challenge* which tests arithmetical ability, problem solving, decision making and reaction speed, and *Memory Matrix* which tests spatial working memory and object recall [27, 28]. These tests were chosen as they are generally found to be more engaging and shorter than more standard psychological reaction speed tasks. In our experience with previous experiments in groups experiencing prolonged isolation, motivation to continue repeated measures experiments, and to maintain a high arousal to generate comparable results at each testing time, declines through the period of isolation (we refer to [18]). Therefore we chose these tasks as participants make more consistent effort to carry them out, and are less likely to withdraw from them during the experimental period. In the *Speed Match* task, the stimuli consisted of symbols presented singly and centrally on the screen and then replaced by another. The participant responded by indicating whether the symbol that appeared matched the previous symbol or not. Following their response the symbol was replaced and the new symbol remained on the screen until the participant responded. Responses were scored based on reaction time, total correct responses and accuracy. In *Chalkboard Challenge*, stimuli consisted of two simple mental arithmetical sums presented on screen side by side. The participant would indicate with a press which sum had the higher value. The game ended after a defined number of inaccurate responses. Stimuli had progressively more complex arithmetical operations. Responses were scored based on total time the participant could continue the game, total number of correct responses, % accuracy and the most difficult level attained. In *Memory Matrix*, tile patterns were presented on the screen for one second. Then they disappeared and the participant indicated on a blank template where the tiles had been. Stimuli became progressively more difficult with larger numbers of tiles to be recalled. The score based on the most difficult level achieved was recorded. Multivariate analysis revealed no results with opposing trend between the three tests and no significant difference (p> .386). For statistical analysis a performance score was calculated as mean out of the scores of the three games for each participant. Data from interval 2 to 7 were calculated as percentage changes from interval 1.

### Statistics

Variables of interest were the factor Group (G_inact_ vs. G_act_) and the factor Interval (intervals 1 to 7). Because there were only four participants in each group, non-parametric tests were used. Mann-Whitney U-test was used for the comparison of the means between the groups. Friedman’s ANOVA was used to compare the seven intervals for each group separately. In case of significance Wilcoxon-test for paired samples was used for the comparison of interval 1 with interval 2 to 7. Bonferroni correction was used.

## Results

### EEG

There was no difference between groups in EEG frequency band alpha (p = .432) or beta at interval 1 (p = .564).

**Fig 2:**
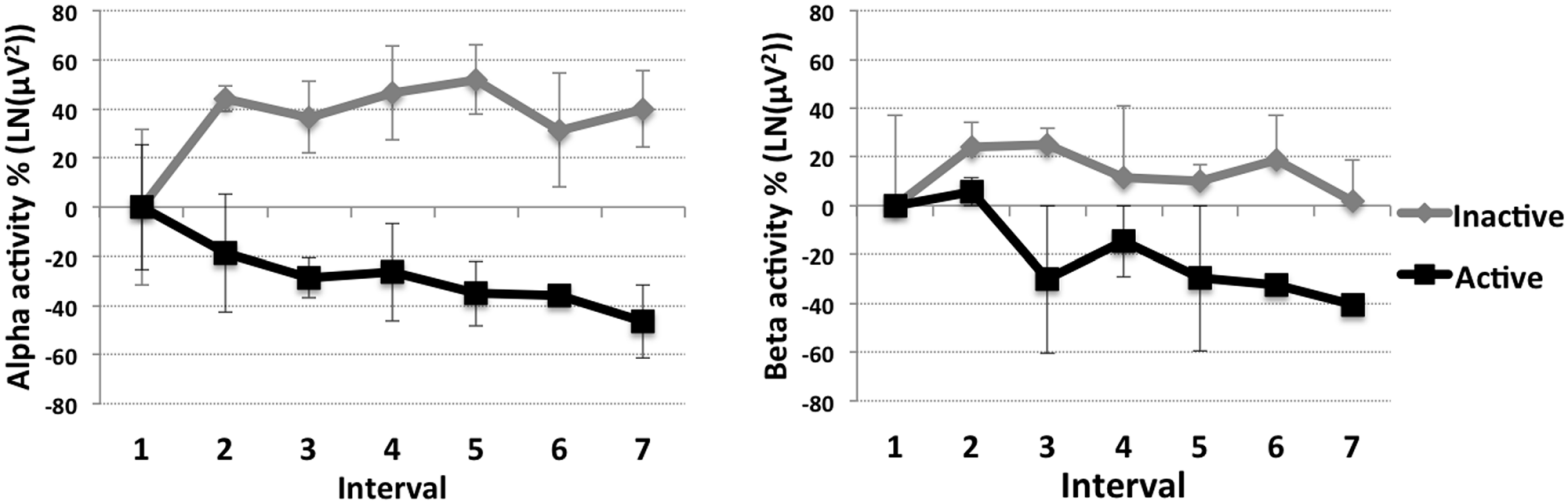
EEG results. Percentage changes from interval 1 of brain activity within the alpha (left) and beta (right) frequency band for the inactive (grey) and the active (black) group in the course of interval 1 to 7 (x-axis). Displayed are mean values plus/minus standard deviations.

In the alpha frequency band, G_inact_ showed higher brain activity through intervals 2 to 7 than G_act_. Significant differences between groups comparing interval 2 to 7 were found (for all comparisons p = .021). No significant changes between intervals were observed (G_inact_: p = .581, G_act_: p = .180) (see Fig 2 and Table 1). Alpha activity of G_inact_ increased from interval 1 to 2 about 44% and then remained high through intervals 2 to 7, whereas for the G_act_ alpha activity progressively decreased over time, to 46% lower at interval 7 compared to interval 1.

**Table 1:** Statistical results.

	Group (MWU-Test)			Interval (Friedman)			Post-hoc (Wilcoxon)		
		Z	p		Chi^2^	p		Z	p^#^
**EEG ALPHA**	Interval 1	0.000	1	Group_inact_	4.714	.581			
	Interval 2	2.309	.021*	Group_act_	8.893	.180			
	Interval 3	2.309	.021*						
	Interval 4	2.309	.021*						
	Interval 5	2.309	.021*						
	Interval 6	2.309	.021*						
	Interval 7	2.309	.021*						
**EEG BETA**	Interval 1	0.577	.564	Group_inact_	6.964	.324	Interval 1		
	Interval 2	1.154	.248	Group_act_	13.929	.030*	Interval 2	0.365	.715
	Interval 3	2.310	.021*				Interval 3	1.826	.068
	Interval 4	1.155	.248				Interval 4	1.826	.068
	Interval 5	2.310	.021*				Interval 5	1.826	.068
	Interval 6	2.310	.021*				Interval 6	1.826	.068
	Interval 7	2.310	.021*				Interval 7	1.826	.068
**STRAIN**	Interval 1	-0.289	.773	Group_inact_	14.778	.022*	Interval 1		
	Interval 2	-2.310	.021*	Group_act_	5.191	.520	Interval 2	1.604	.109
	Interval 3	-2.310	.021*				Interval 3	1.826	.068
	Interval 4	-2.310	.021*				Interval 4	1.826	.068
	Interval 5	-2.310	.021*				Interval 5	1.826	.068
	Interval 6	-2.310	.021*				Interval 6	1.826	.068
	Interval 7	-2.310	.021*				Interval 7	1.826	.068
**MOT**	Interval 1	-0.289	.773	Group_inact_	8.177	.225			
	Interval 2	-2.310	.021*	Group_act_	4.218	.647			
	Interval 3	-2.310	.021*						
	Interval 4	-2.021	.043*						
	Interval 5	-2.310	.021*						
	Interval 6	-2.310	.021*						
	Interval 7	-2.310	.021*						
**PEPS**	Interval 1	-0.289	.773	Group_inact_	9.855	.131			
	Interval 2	-2.021	.043*	Group_act_	2.973	.812			
	Interval 3	-2.310	.021*						
	Interval 4	-2.310	.021*						
	Interval 5	-2.310	.021*						
	Interval 6	-2.310	.021*						
	Interval 7	-2.310	.021*						
**BRAIN GAMES**	Interval 1	0.289	.773	Group_inact_	6.750	.345			
	Interval 2	0.289	.773	Group_act_	6.964	.324			
	Interval 3	0.577	.564						
	Interval 4	-0.866	.386						
	Interval 5	0.866	.386						
	Interval 6	0.289	.773						
	Interval 7	0.000	1						

^#^Footnote: For Wilcoxon Test, p< .0083 would be the level of significance after Bonferroni correction.

* Significance level p< .05.

For the beta frequency band, G_inact_ showed higher beta activity than G_act_ comparing interval 2 to 7. A significant effect of group for interval 3, 5, 6 and 7 was revealed (for all p = .021). No differences between intervals for G_inact_ were found (p = .324), but for G_act_ beta activity progressively decreased up to 40% at interval 7 (p = .030).

### Mood

There was no difference in mood state at interval 1 for any of the three dimensions (STRAIN: p = .681, MOT: p = .560, PEPS: p = .574).

**Fig 3:**
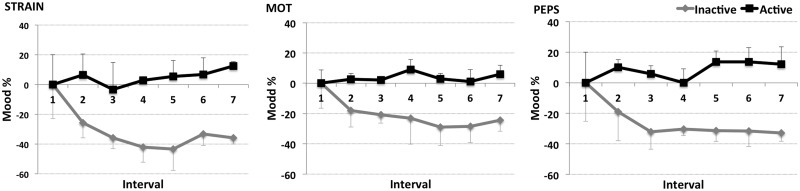
Mood results. Percentage changes from interval 1 of the dimension perceived psychological strain (left), perceived motivational state (middle), and perceived physiological state (right) for the inactive (grey) and the active (black) group in the course of interval 1 to 7 (x-axis). The bar graphs on the right show the mood values at interval one for both groups. Displayed are mean values plus/minus standard deviations.

For STRAIN, the groups were significantly different for intervals 2 to 7 (for all comparisons p = .021, see Fig 3 left and Table 1). G_act_ showed no effect of interval (p = .520), whereas G_inact_ developed a significant change (p = .022) which turned out to be a negative progression over the intervals, meaning that perceived psychological strain decreased up to 43% at interval 5.

A significant group effect was found for MOT comparing intervals 2 to 7 respectively (for interval 2, 3, 5, 6 and 7 p = .021, for interval 4 p = .043), for which the G_act_ showed higher ratings than the G_inact_ respectively (see Fig 3 middle). No effect for interval was found (for G_inact_: p = .225, for G_act_: p = .647), although G_inact_ showed a negative trend of perceived motivational state over time, falling to 28% lower at intervals 5 and 6.

Regarding PEPS, a group effect for interval 2 to 7 was found (interval 2 p = .043, interval 3 to 7 p = .021) with higher ratings for G_act_. G_act_ and G_inact_ did not show significant changes over the intervals (G_act_ p = .812; G_inact_ p = .131; see Fig 3 right).

### Cognitive Tasks

**Fig 4:**
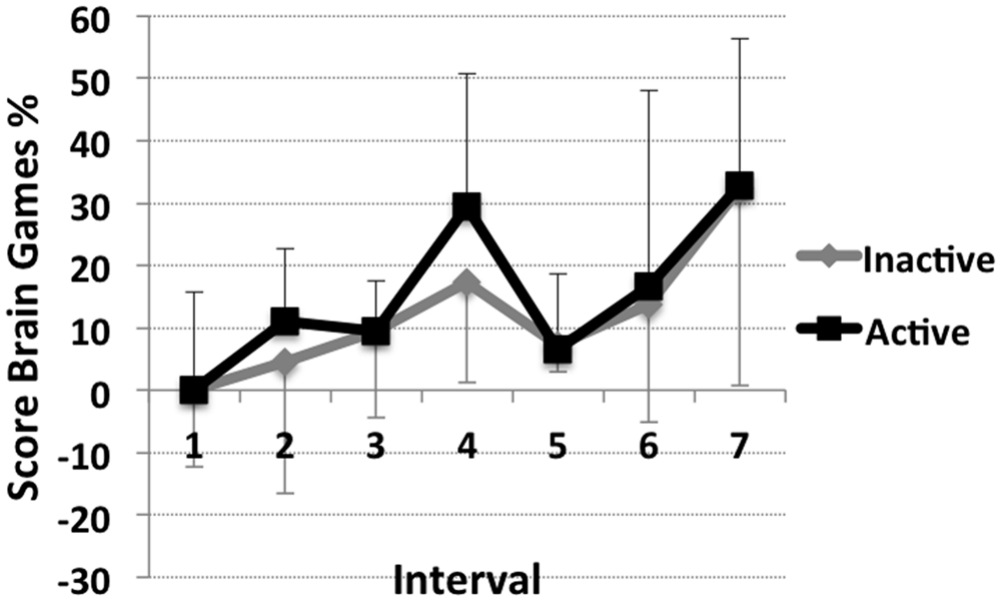
Results Cognitive tasks. Percentage changes from interval 1 of Brain Games Score (mean of the three games) for the inactive (grey) and the active (black) group in the course of interval 1 to 7 (x-axis). Displayed are mean values plus/minus standard deviations.

Within the cognitive tasks, no difference was observed between groups (p> .386), tests (p> .386) or between intervals (G_inact_ p = .345; G_act_ p = .324; see Fig 4 and Table 1). No interaction between task, interval and group were found.

## Discussion

The study aimed to investigate the effect of long-term isolation on human brain activity, mood and cognitive performance, and—in this regard—the influence of exercise on the negative sequelae associated with prolonged isolation in a confined, hostile environment. A long-term effect of exercise was observed for mood, such that regularly active people showed a steady mood, and a decrease in alpha and beta brain activity in the course of isolation. Brain cortical activity decreased concurrently. Inactive people, in contrast, showed a deterioration of mood, and no significant changes in brain activity. The cognitive tests used in our study did not show any influence of isolation or exercise.

### EEG

Activity within the alpha and beta frequency range showed a parallel response to physical activity and isolation over the winter-over in both G_inact_ and G_act_. In keeping with our results, numerous recent studies have demonstrated parallel changes within the alpha and beta frequency band [7, 23, 24], contradicting the traditional theory of cortical arousal. Thus, it seems that the traditional model of cortical arousal [22], in which alpha increases and beta decreases with relaxation, or vice versa for arousal, does not appear to hold true. It may be more appropriate to interpret this parallel behavior found in our study as generally higher or lower brain cortical activity [7].

Activity in the brain’s cortex clearly differentiated the both groups. For G_inact_, cortical activity slightly increased from interval 1 to interval 2. These intervals reflect the transition from the busy and crowed summer to the more monotone and lonely winter campaign at Concordia Station. The work in the winter period is reduced to what is possible and necessary to operate and live at the Station. Thus, daily life during the winter-over is affected by less variety from there on. In addition, communication and social contact is limited to the small group of colleagues on site, and, during winter 2011, to restricted email and phone conversations to the outer world. Accordingly, G_inact_ seems to respond to these constrictions with consistent, even slightly elevated brain activity (alpha and beta), which, we propose, indicates a constant/elevated level of cortical arousal [7]. Throughout the course of the winter, G_inact_ remained on this ‘elevated’ level of activation. G_act_ instead showed a decreasing trend of activity within the alpha and beta frequency bands. Similar findings have been found the MARS105 mission, a laboratory isolation study. Schneider et al. [7] demonstrated progressively decreasing activity within the alpha and beta frequency bands within the six participants, in the course of their 105-day isolation. They attributed this parallel change of alpha and beta to an overall decrease in brain activation state due to the prolonged isolation. Our results suggest a more complex picture otherwise a similar pattern should have been observed for both, G_inact_ and G_act_. The different progressions of the brain activation state rather appear to be related to the more or less active life-style of the groups.

### Mood

At the beginning of the winter-over, there was no difference in self-reported mood between G_act_ and G_inact_. Right from the beginning, G_act_ exercised at a higher intensity and frequency. The exercise patterns of the groups remained very stable throughout the period of isolation. The inactive group (G_nact_) experienced a progressive deterioration in motivation, and perceived physical state, as well as decreasing psychological strain that evolved over the period of isolation. Therefore, given that the active group, G_act_, did not experience these declines, we argue that our data has demonstrated that voluntary exercise with a certain intensity (>2500 arbitrary training units/interval) positively affects mood in term of motivation and perceived physical state, in isolated, confined environments.

The favorable effect of active living on mood is a well-known and investigated phenomenon [29]. Because of the known benefits that exercise has on mood, exercise interventions are a recognized method for prevention and therapy for mental disorders [30–33]. Therefore our results are in keeping with the literature based on non-isolated groups.

The EEG results are at least partially consistent with previous studies (11). We speculate that the decreasing brain activation state of G_act_ reflects an adaptation to the isolation, to cope with the stimuli-reduced environment [34]. As a consequence, G_act_ did not demonstrate any mood impairments. By contrast, G_inact_ experienced increased brain activity at the beginning, and did not experience the depression in cortical activity experienced by G_act_ in the course of isolation. This might be a result of accumulating stress, without an appropriate outlet, such as exercise, to ameliorate their cumulative mood impairments [34, 35].

In our study neither group showed a seasonal progression of mood as observed in other polar and isolation studies, such as a decrease of mood in the first and second half of the isolation period with a positive peak at mid point and positive trend towards the end of isolation [7, 36]. It has been observed that differences between individual expeditions, their location, crew composition, and the progress of the isolation lead to different developments of these symptoms, making it impossible to predict the outcome in advance [36]; which is a major problem for isolation and space mission preparation.

Nevertheless, the negative progressions of mood of the G_inact_ and the steady mood of G_act_ indicate a positive long-term effect of exercise. This effect can mainly be traced back to the voluntary training sessions with self-selected exercise. The assumption is based on publications stating that processes related to familiarizations and adaptations to exercise are important factors for the exercise response [17, 37–39]. These processes deal with the combination of perceived exertion, individual abilities, goals and preferences, plus the perceived autonomy during the activity [17, 37–39]. In short, the more comfortable, secure and self-determined the exerciser feels, the higher the positive outcome seems to be. This is in line with the results of the MARS500 study, in which six crewmembers isolated for 520 days showed the most considerable exercise response following their preferred exercise session [18]. At the same time, certain exercise intensity seems to be needed, because low-intensity exercise did not reveal an effect on brain cortical activity in a former study [37] and also the one subject who exercised with a similar frequency but lower intensity did not show the positive effect. Accordingly, based on our results we would recommend regularly performed self-determined but ambitious exercise to individuals in isolated, confined environments in order to maintain mood.

### Cognition

No significant changes of cognitive performance with isolation or between groups were identified. Both groups showed a non-significant trend toward improvement over time with a short-term peak at interval 4 (mid-point). This progression can probably be attributed to learning, because both groups improved their score to approximately the same degree (interval 7 compared to interval 1: G_inact_: 32.3%; G_act_: 32.7%). All participants were subject to a familiarization session prior to the first interval in order to avoid measurement errors due to novelty of the task. But, familiarization did not aim to complete learning processes.

Cognitive impairments with duration of isolation were expected. The recognized winter-over syndrome includes a reversible decline in cognition [6]. However, not all Antarctic studies observed cognitive impairments [40, 41] and not all winter crews develop the winter-over syndrome. All our tests examined low-level visuospatial and mathematical, cognitive functions. It may be that the tasks involved functions that remained unaffected, that the tests were not sufficiently sensitive to detect changes.

### Remarks

A recognized possible problem with the study is that the energy expenditure during working hours may have been highly variable between individuals, which could have influenced time spent exercising after work. Nevertheless, although the sample size of this study is small, our results suggest that voluntary, self-determined exercise of adequate intensity in isolation can be beneficial for crewmembers mood. However, we need to regard the results with caution because of the small sample size.

The current study underscores the need for further investigation into the effect of exercise and its potential to protect and support psycho-physiological health.

## Conclusion

Our results demonstrate the positive effect of regularly performed voluntary exercise, which supports subjective mental well-being of people in long-term isolation. The results are not only of interest for future isolation and space missions, but also for our increasingly inactive and aging population.
